# A biosafety level-2 dose-dependent lethal mouse model of spotted fever rickettsiosis: *Rickettsia parkeri* Atlantic Rainforest strain

**DOI:** 10.1371/journal.pntd.0007054

**Published:** 2019-06-19

**Authors:** Andrés F. Londoño, Nicole L. Mendell, David H. Walker, Donald H. Bouyer

**Affiliations:** 1 Department of Pathology, The University of Texas Medical Branch, Galveston, Texas, United States of America; 2 Research Group on Veterinary Sciences “Centauro”, Facultad de Ciencias Agrarias, Universidad de Antioquia, Medellín, Antioquia, Colombia; Instituto de Pesquisas Veterinarias Desiderio Finamor, BRAZIL

## Abstract

**Background:**

The species of the *Rickettsia* genus are separated into four groups: the ancestral group, typhus group, transitional group and spotted fever group. *Rickettsia parkeri*, a spotted fever group *Rickettsia*, has been reported across the American continents as infecting several tick species and is associated with a relatively mild human disease characterized by eschar formation at the tick feeding site, regional lymphadenopathy, fever, myalgia and rash. Currently, there are several mouse models that provide good approaches to study the acute lethal disease caused by *Rickettsia*, but these models can only be performed in an animal biosafety level 3 laboratory. We present an alternative mouse model for acute lethal rickettsial disease, using *R*. *parkeri* Atlantic Rainforest strain and C3H/HeN mice, with the advantage that this model can be studied in an animal biosafety level 2 laboratory.

**Principal findings:**

In the C3H/HeN mouse model, we determined that infection with 1x10^6^ and 1x10^7^ viable *R*. *parkeri* Atlantic Rainforest strain organisms produced dose-dependent severity, whereas infection with 1x10^8^ viable bacteria resulted in a lethal illness. The animals became moribund on day five or six post-infection. The lethal disease was characterized by ruffled fur, erythema, labored breathing, decreased activity, and hunched posture, which began on day three post-infection (p.i.) and coincided with the peak bacterial loads. Significant splenomegaly (on days three and five p.i.), neutrophilia (on days three and five p.i.), and thrombocytopenia (on days one, three and five p.i.) were observed.

**Significance:**

Since *R*. *parkeri* is used at biosafety level 2, the greatest advantage of this inbred mouse model is the ability to investigate immunity and pathogenesis of rickettsiosis with all the tools available at biosafety level 2.

## Author summary

*Rickettsia* is a bacterial genus that contains distinct species that are transmitted by arthropods. Many of these agents produce infection and disease in humans. The illness can range from very severe, such as Rocky Mountain spotted fever caused by *Rickettsia rickettsii* to mild human disease characterized by eschar formation at the tick feeding site and less severe symptoms caused by *Rickettsia parkeri* and often apparently asymptomatic seroconversion as observed with *R*. *amblyommatis*. To study these diseases, animal models are invaluable, and mouse models offer the best advantages for studies of immunity and pathogenesis because of the availability of immunologic reagents and gene knockout animals. Several mouse models are available for the study of the acute lethal disease produced by these bacteria, providing the opportunity to test different treatments and vaccine candidates. However, work with these models requires an animal biosafety level 3 laboratory. In this report, we present an alternative mouse model with *R*. *parkeri* Atlantic Rainforest strain available for investigation in a biosafety level 2 laboratory to study an acute dose-dependent lethal spotted fever group rickettsial disease with the advantage that experiments can be performed at this biosafety level.

## Introduction

*Rickettsia*, a genus of Alphaproteobacteria that contains microorganisms transmitted by arthropods, is divided into four groups: the ancestral group (e.g., *R*. *bellii*), typhus group (e.g., *Rickettsia typhi*), transitional group (e.g., *R*. *australis*) and spotted fever group (SFG) (e.g., *R*. *conorii* and *R*. *parkeri*) [1,2].

*Rickettsia parkeri*, a member of the SFG, has been reported across the American continents as infecting several tick species including the *Amblyomma maculatum* complex (*A*. *maculatum*, *A*. *triste* and *A*. *tigrinum*), *A*. *ovale* complex (*A*. *ovale* and *A*. *aureolatum*), *A*. *nodosum*, *A*. *parvitarsum* and *Dermacentor parumapertus* [3,4]. Even though four *R*. *parkeri* strains have been described, until now only *R*. *parkeri* sensu stricto and *R*. *parkeri* Atlantic Rainforest strain (ARF) are considered pathogenic to humans. On the other hand, *R*. *parkeri* rickettsiosis (disease caused by *R*. *parkeri*) is the second most important tick-borne rickettsiosis in USA, after *R*. *rickettsii* rickettsiosis [5,6]. Cases have been described in Argentina and Uruguay [7,8]. *Rickettsia parkeri* ARF strain also causes eschar-associated rickettsiosis in Brazil and is associated with *A*. *ovale* ticks as the principal vector and probably with *A*. *aureolatum* ticks as an alternative vector [9–13]. The Colombian *R*. *parkeri* isolate ARF is phylogenetically the same based on the available sequences for five protein-encoding genes of the Brazilian RpARF strain, which was also isolated from *A*. *ovale* ticks (a recognized vector in Brazil) [10,14] and closely related to the Black Gap strain isolated from a *Dermacentor parumapertus* collected in Texas [3]. The Colombian and Brazilian isolates of RpARF also share conserved sequences in two of the three published intergenic spacers [4, Londono et al; manuscript in preparation]. *Rickettsia parkeri* has been associated with a relatively mild human disease characterized by eschar formation at the tick feeding site, regional lymphadenopathy, fever, myalgia and rash [12,13,15].

Previously described mouse models provide a good approach to study the acute lethal disease produced by *Rickettsia*, such as *R*. *australis* infection of C57BL/6 or Balb/c mice, *R*. *conorii* infection of C3H/HeN mice and *R*. *typhi* infection of C3H/HeN mice [16–18]. The first one can be utilized to study a highly invasive model of rickettsial disease, because infection involves not only endothelial cells, but also perivascular cell types such as macrophages and can utilize gene knockout mice on the C57BL/6 background [16]. The second mouse model, *R*. *conorii* infection of C3H/HeN mice, is the best available model for SFG rickettsial diseases, because the principal target cells are the vascular endothelium [17]. The third one, *R*. *typhi* infection of C3H/HeN mice, is useful to study immunity and pathogenesis in typhus group rickettsial infection [18], but all of the aforementioned models are limited to BSL3 use only.

Previous animal model publications, where *R*. *parkeri* was used as infectious agent, did not state the biosafety level used [19,20], but it is well accepted that the researchers use this agent at biosafety level 2 containment [21]. The aim of this study was to characterize the pathogenicity of RpARF and describe the clinical course, outcome, pathologic lesions, and anatomic distribution of rickettsiae in this new mouse model of SFG rickettsiosis that can be performed at biosafety level 2.

## Materials and methods

### Rickettsial quantification

A low-passage stock of RpARF isolated from a Colombian *A*. *ovale* tick and grown in Vero cells was used for animal experiments [14]. This stock was passaged eight times in Vero cells and once in C3H/HeN mice to remove *Mycoplasma* sp. contamination. Rickettsial identity and *Mycoplasma* sp. removal were confirmed by full genome MinION nanopore and Illumina sequencing. The infected Vero cells were harvested and then frozen in sucrose-phosphate-glutamate buffer solution (218 mM sucrose, 3.76 mM KH_2_PO_4_, 7.2 mM K_2_HPO_4_, and 3.9 mM glutamate). Rickettsial quantification was performed using a qPCR assay to quantify the viable bacteria. Briefly, a six-well plate with confluent Vero cells was inoculated with 1 ml of RpARF stock at a 1:100 dilution in MEM with 1% bovine calf serum (BCS). The medium was aspirated from each well, and the wells were infected in triplicate or left as negative controls. The plate was centrifuged at 730 RCF for five minutes and incubated at 37°C for one hour. After one hour incubation to allow for rickettsial adsorption and entry into the cells, the plate was washed three times with warm (37°C) sterile PBS to remove extracellular rickettsiae and incubated again for one hour with 1 ml of 1% BCS growth medium with DNase (10U/μl) to degrade DNA of non-viable rickettsiae. The plate was washed three times followed by DNA extraction using the DNeasy Blood and Tissue Kit (Qiagen, Hercules, CA). For this purpose, 200 μl of PBS and 200 μl of the buffer AL were added to each well and incubated for 2 minutes. The contents of each well were collected in a clean tube and incubated with 20 μl of proteinase K at 56°C for 10 minutes. A positive control consisted of DNA from 10 μl of the original stock with 190 μl of PBS that was extracted in parallel. The DNA was further extracted following the manufacturer’s protocol. Primers for the single copy *gltA* gene, CS-5 (GAGAGAAAATTATATCCAAATGTTGAT) and CS-6 (AGGGTCTTCGTGCATTTCTT), were used for quantitative real-time PCR (qPCR) [22] with iTaq Universal SYBR Green Supermix (Bio-Rad, Hercules, CA), and the standard curve was prepared with dilutions (10^9^ to 10^1^ copies/μl) of a *R*. *conorii gltA* PCR fragment-containing plasmid, adapted from the assay used for *Orientia* [23]. The concentration of the RpARF stock used for this study was determined to be 3.72 x 10^9^ viable bacteria per ml. This value was utilized to calculate the doses of rickettsiae (1 x 10^8^, 1 x 10^7^ or 1 x 10^6^ bacteria) in a total volume of 200 μl.

### Mice

Eight week old male C3H/HeN mice from Charles River Laboratories, Inc. (Houston, TX) were used in this study. Since the RpARF was originally isolated at BSL-3, by the requirements of the Institutional Biosafety Committee the experiments were performed in an animal biosafety level 3 facility, under specific pathogen-free conditions. All animal work was approved by the Institutional Animal Care and Use Committee (protocol # 9007082) of the University of Texas Medical Branch-Galveston, and mice were used according to the guidelines in the Guide for the Care and Use of Laboratory Animals and comply with the USDA Animal Welfare Act (Public Law 89–544), the Health Research Extension Act of 1985 (Public Law 99–158), the Public Health Service Policy on Humane Care and Use of Laboratory Animals, and the NAS Guide for the Care and Use of Laboratory Animals (ISBN-13).

### Preliminary dose range experiment

To characterize the pathogenicity of our isolate of RpARF in a murine model, a dose range-finding experiment was undertaken. Groups of mice (n = 3/group) were inoculated intravenously (I.V.) with 1x10^6^, 1x10^7^ or 1x10^8^ viable bacteria or PBS (200 μl). Animals were monitored daily for signs of illness, and rectal temperature and body weight were measured until moribund or the survivors were sacrificed at day 14 post infection.

### Bacterial load assays

To characterize the kinetics of the lethal murine model of infection with RpARF, three groups of animals (n = 6/group) were infected with 200 μl of the highest dose (1x10^8^) I.V., and one group was utilized as controls (200 μl of PBS I.V.). Due to the kinetics of the high dose infection, which resulted in a lethal moribund state by day six p.i., infected mice were sacrificed on days one, three and five post-infection (p.i.), and the animals from the control group (n = 6) were sacrificed on day six. As in the first study, the animals were monitored daily. The spleen, kidneys, liver, lungs, heart and brain were collected from each animal to determine bacterial loads and evaluate histopathologic changes. We collected blood samples in BD microtainer tubes with and without K_2_EDTA (Becton Dickinson, Franklin Lakes, NJ) to analyze the blood cell counts, determine bacterial loads, and assess the serologic response.

### Hematology and serology

Blood samples were collected in two microtainer tubes, with and without K_2_EDTA, from each animal at the time of sacrifice. The complete blood cell counts were measured with a HemaVet 950FS apparatus (Drew Scientific, Miami Lakes, FL). Indirect immunofluorescence assay (IFA) was performed to measure IgG and IgM antibodies to RpARF using acetone-permeabilized RpARF-infected Vero cell-coated slides. The slides were immersed in phosphate buffered saline (PBS) for 10 minutes at room temperature, transferred to blocking solution (PBS with 1% bovine serum albumin [BSA] and 0.01% sodium azide), and incubated for 15 minutes. Sera were diluted in a series of two-fold dilutions starting at 1:64 in a solution of PBS with 1% BSA and 0.1% Tween-20. Experimental samples of serum as well as one positive control and one negative control serum per slide were added to the wells and incubated at 37°C for 30 minutes in a humidified chamber. For IgM titer determination, IgG binding was blocked by treating the serum with IgM Pretreatment Diluent (Focus Diagnostics, California, USA) prior to assaying. The slides were washed two times for 10 minutes in PBS containing 0.1% Tween-20. Secondary antibody, DyLight 488-conjugated anti-mouse IgG (1:15,000 dilution, Jackson Immunoresearch, West Grove, PA) or FITC-conjugated anti-mouse IgM antibody, mu-chain specific (1:500 dilution, Vector Laboratories, Burlingame, CA), was added to the wells and incubated for 30 minutes in a humidified chamber. Finally, the slides were washed twice as before with the final wash containing 1% Evans blue solution, mounted with DAPI fluoromount-G (SouthernBiotech, Birmingham, AL), and coverslipped. Slides were observed under a fluorescence microscope at 400X magnification (Olympus Scientific, Waltham, MA).

### Measurement of rickettsial loads by real-time PCR

DNA was extracted from tissue and blood using a Qiagen DNeasy Blood and Tissue Kit (Qiagen, Hercules, CA), following the manufacturer’s protocol. The bacterial loads were determined by qPCR using the primers CS-5 and CS-6 with the probe (FAM—CATTGTGCCATCCAGCCTACGGT), and iQ Supermix (Bio-Rad, Hercules, CA) [22,24]. The standard curve was determined as described above in a qPCR assay to measure the viable bacteria. Samples were normalized using tissue weight or blood volume, and the concentration of rickettsiae is expressed as gene copies per milligram of tissue or milliliter of blood.

### Histology and immunohistochemistry

The tissues were fixed with 10% neutral buffered formalin for two weeks and embedded in paraffin. The samples were sectioned at 5 μM thickness and stained with hematoxylin and eosin for histological analysis or processed for immunohistochemical (IHC) staining of rickettsial antigen [19].

The sections were incubated at 54°C overnight, deparaffinized and hydrated. After that, they were blocked with Avidin/Biotin Blocking Kit (Life Technologies, Frederick, MD) and treated with proteinase K (Dako, Carpinteria, CA), for antigen retrieval. The sections were incubated with polyclonal rabbit anti-*R*. *conorii* antibody (1:300 dilution, produced in-house) at room temperature for one hour, followed by biotinylated secondary anti-rabbit IgG (1:200 dilution, Vector Laboratories, Burlingame, CA), streptavidin-AP (1:200, Vector Laboratories, Burlingame, CA) for 30 minutes each and Fast Red (Dako, Carpinteria, CA) for 5 minutes. The sections were washed twice with Tris-buffered saline containing 0.05% Tween-20. Slides were counterstained with hematoxylin, dehydrated, mounted with Permount and examined with an Olympus BX51 microscope (Olympus Scientific, Waltham, MA).

### Statistical analysis

Data were analyzed using GraphPad Prism software utilizing a one-way ANOVA with Tukey’s post-test. P-values of <0.05 were considered to indicate statistically significant differences in each analysis.

## Results

### Susceptibility and lethal dose of RpARF in a murine model

**Fig 1:**
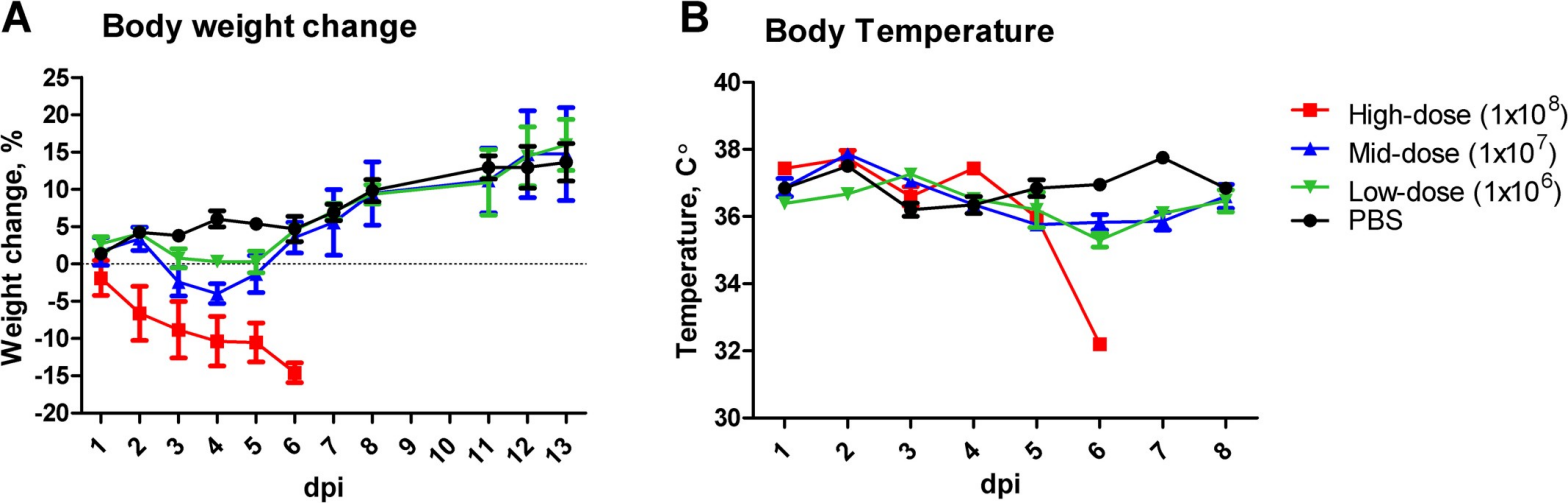
Kinetics of body weight (**A**) and temperature changes (**B**) in C3H/HeN mice infected intravenously with high- (1x10^8^, square), mid- (1x10^7^, triangle) and low- (1x10^6^, inverted triangle) doses of viable RpARF or PBS inoculated controls (circle).

**Fig 2:**
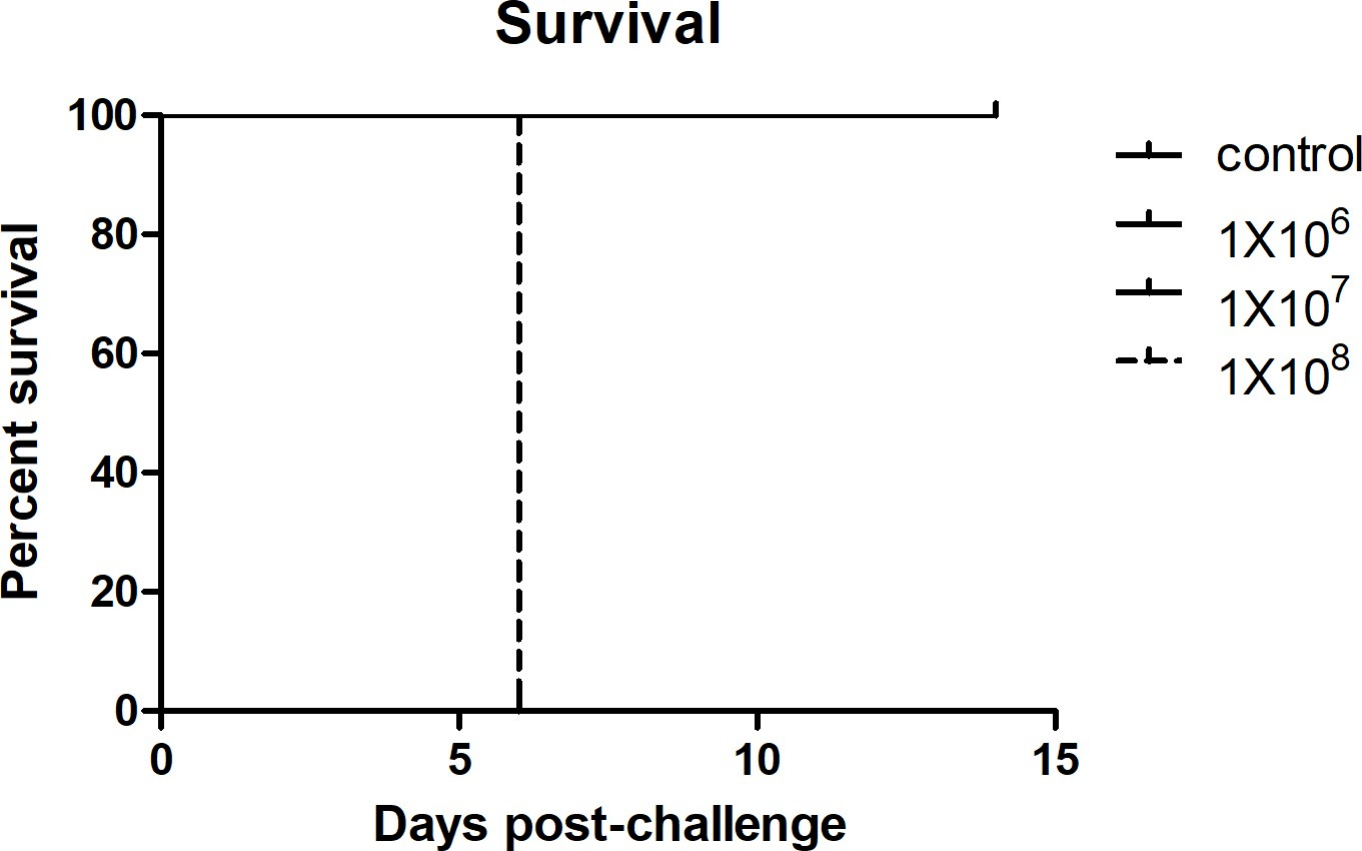
Survival curve of mice following challenge with 1x10^6^, 1x10^7^, or 1x10^8^ viable RpARF or PBS.

In C3H/HeN mice, we observed that infection with 1x10^6^ (low dose) and 1x10^7^ (mid dose) of viable *R*. *parkeri* ARF produced dose-dependent severity of illness. The animals which received the lowest concentration doses began to show signs of illness on day three p.i. as evidenced by onset of weight-loss. Animals in the lowest dose group presented with piloerection on days three and four p.i.; whereas piloerection on day three, and piloerection, erythema, and hunched posture were observed on day four in the mid dose group, which diminished to piloerection alone by five days p.i. By days five and six p.i., the mice of the low- and mid-dose groups, respectively, did not have observable signs of illness through the experiment’s end (day 14). The control group did not present with any sign of illness during the experimental observation period. Infection with 1x10^8^ (high dose) of viable bacteria resulted in a lethal illness. The animals became moribund on day six p.i., when the mice had lost 14.6% of their initial body weight and developed hypothermia (Figs 1A, 1B and 2). From this point, all investigations were performed with the lethal dose.

### Kinetics of signs of illness in the lethal murine model of infection with RpARF

**Fig 3:**
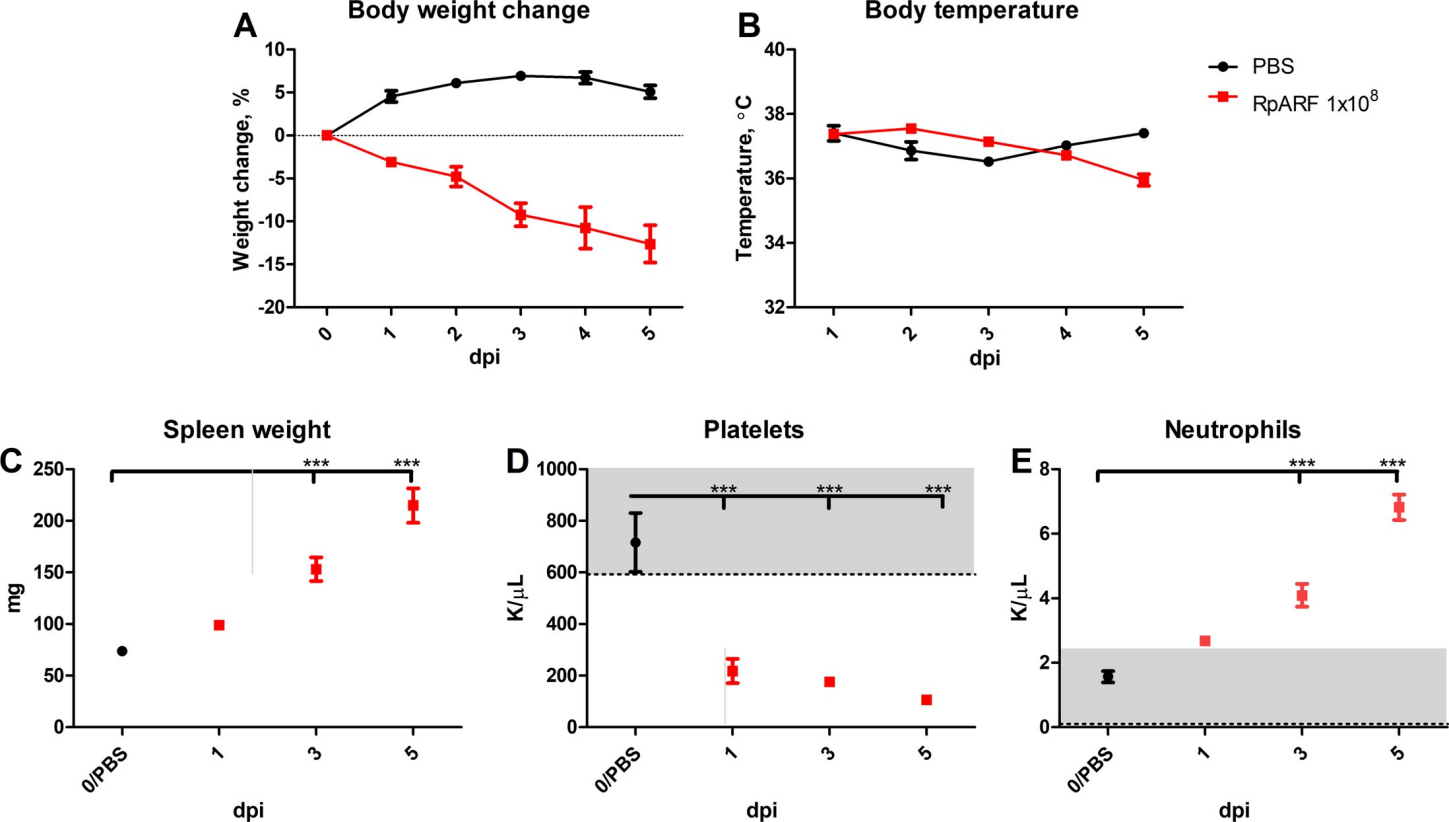
Kinetics of changes in weight (**A**), body temperature (**B**), and spleen weight (**C**) of mice infected intravenously with 1x10^8^ viable RpARF organisms and uninfected controls. Mean platelet (**D**) and mean neutrophil concentrations (**E**) of mice infected intravenously with 1x10^8^ viable RpARF organisms on days 1, 3 and 5 p.i. and uninfected controls measured with a HEMAVET 950FS apparatus.

The disease was characterized by ruffled fur, erythema, labored breathing, decreased activity, and hunched back, which began on day three p.i., and the severity of these signs intensified with time. The decreased body weight and body temperature observed in the high dose recipients in the dose-range study were replicated (Fig 3A and 3B). Significant splenomegaly was observed on days three and five when compared with the control group (Fig 3C).

### Hematology and serology

The most marked hematologic findings of the lethally infected animals were significant thrombocytopenia on days one, three and five p.i. that gradually progressed in severity each day and neutrophilia on days three and five p.i. (Fig 3D and 3E). The animals infected with the low- and mid-doses developed a strong antibody response measured by IFA on day 14 p.i. The IgM reciprocal endpoint titers were between 1,024 and 4,096 for both groups and the IgG titers between 8,192 and 16,384 for the animals that received the low dose, and 16,384 and 32,768 for the animals that received the mid dose. In the animals that received the high dose, IgM antibody was detectable on day three with reciprocal endpoint titers between 256 and 512, and between 512 and 1,024 on day five. In contrast, IgG antibody was present in only two of the six mice on day five at the cutoff reciprocal titer of 64. The serologic results from all animals from the control groups in preliminary dose range experiment and infection experiments were negative.

### Measurement of rickettsial loads by real-time PCR

**Fig 4:**
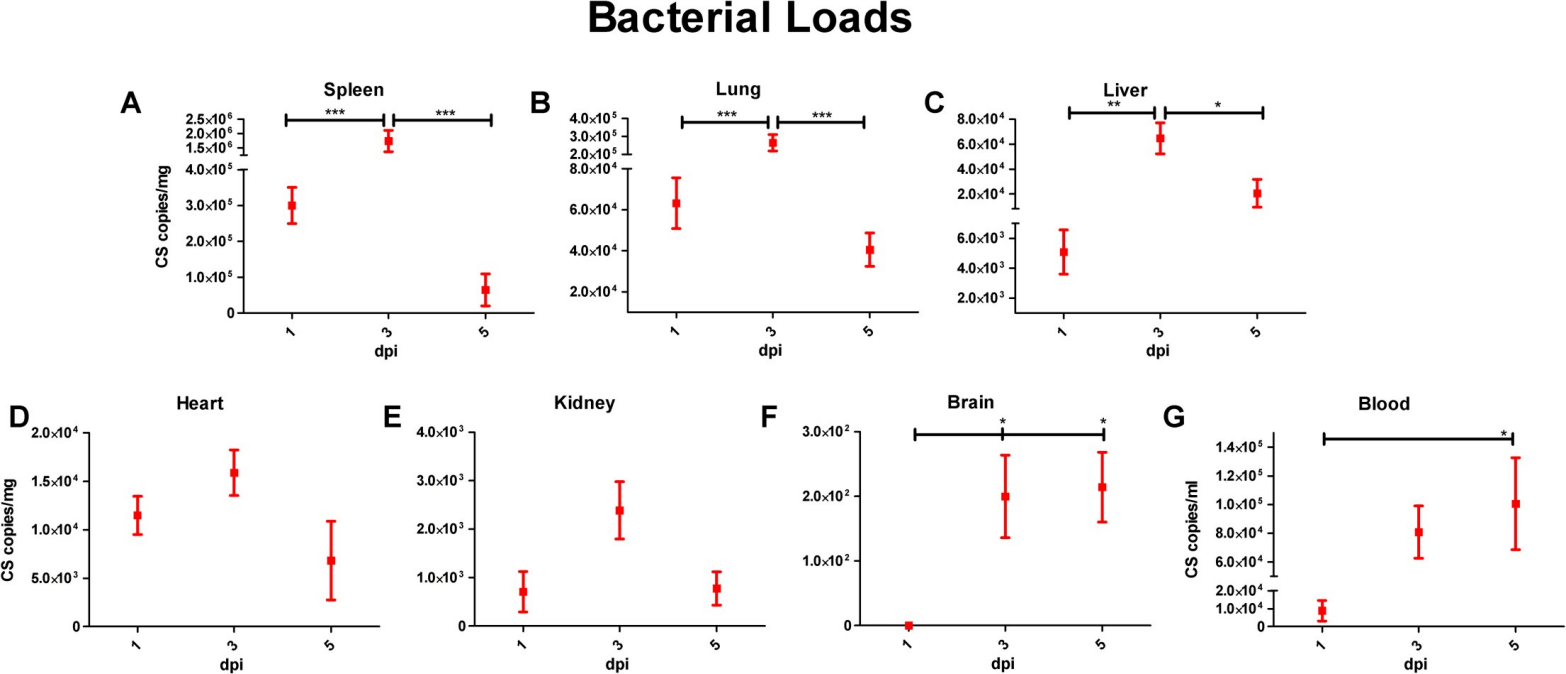
Measurement of rickettsial loads in spleen (**A**), lung (**B**), liver (**C**), heart (**D**), kidney (**E**), brain (**F**), and blood (**G**) expressed as copies of citrate synthase (CS) gene per milligram of tissue or per milliliter of blood on days 1, 3 and 5 p.i. of mice infected with 1x10^8^ viable RpARF organisms.

All tissues of RpARF-infected mice infected with the lethal dose contained rickettsial DNA. In the spleen, lung and liver, qPCR analysis revealed that the peak bacterial loads occurred on day three p.i., with statistical significance, and decreased on day five p.i. (Fig 4A, 4B and 4C). In the heart and kidney, the bacterial loads appeared to peak on day three, but without differences among the days of infection reaching statistical significance (Fig 4D and 4E). Infection was observed in the brain on days three and five p.i., with no statistical difference between the time points (Fig 4F). In the blood, the peak bacterial load was observed on day five p.i. (Fig 4G). All tissues from the control group were negative.

### Histology and immunohistochemistry

**Fig 5:**
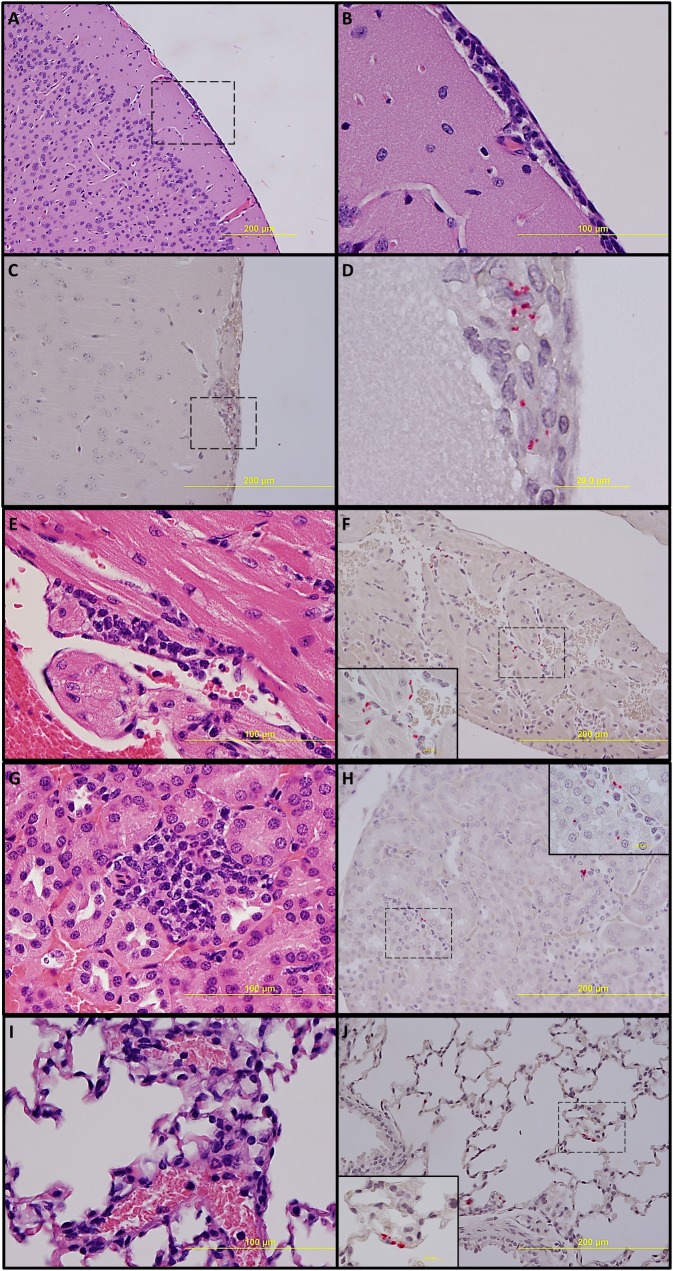
Pathologic lesions and IHC demonstration of SFG rickettsiae in brain: Meningitis on day 3 p.i. (**A**, 100X) and inset (**B**, 400X). Meningitis with presence of rickettsiae (red) (**C**, 200X) and inset (**D**, 1,000X) on day 5 p.i. In heart: Endocarditis and perivascular interstitial inflammation on day 3 p.i. (**E**, 400X). Endothelial presence of rickettsiae (red) (**F**, 200x) and inset (1,000X) on day 3 p.i. In kidney: Inflammation with interstitial mononuclear cellular infiltration on day 3 p.i. (**G**, 400X). Endothelial presence of rickettsiae (red) (**H**, 200X) and inset (1,000X) on day 3 p.i. In lung: Interstitial pneumonia on day 3 p.i. (**I**, 400X). Endothelial presence of rickettsiae (red) in alveolar capillaries (**J**, 200X) and inset (1,000X) on day 3 p.i.

**Fig 6:**
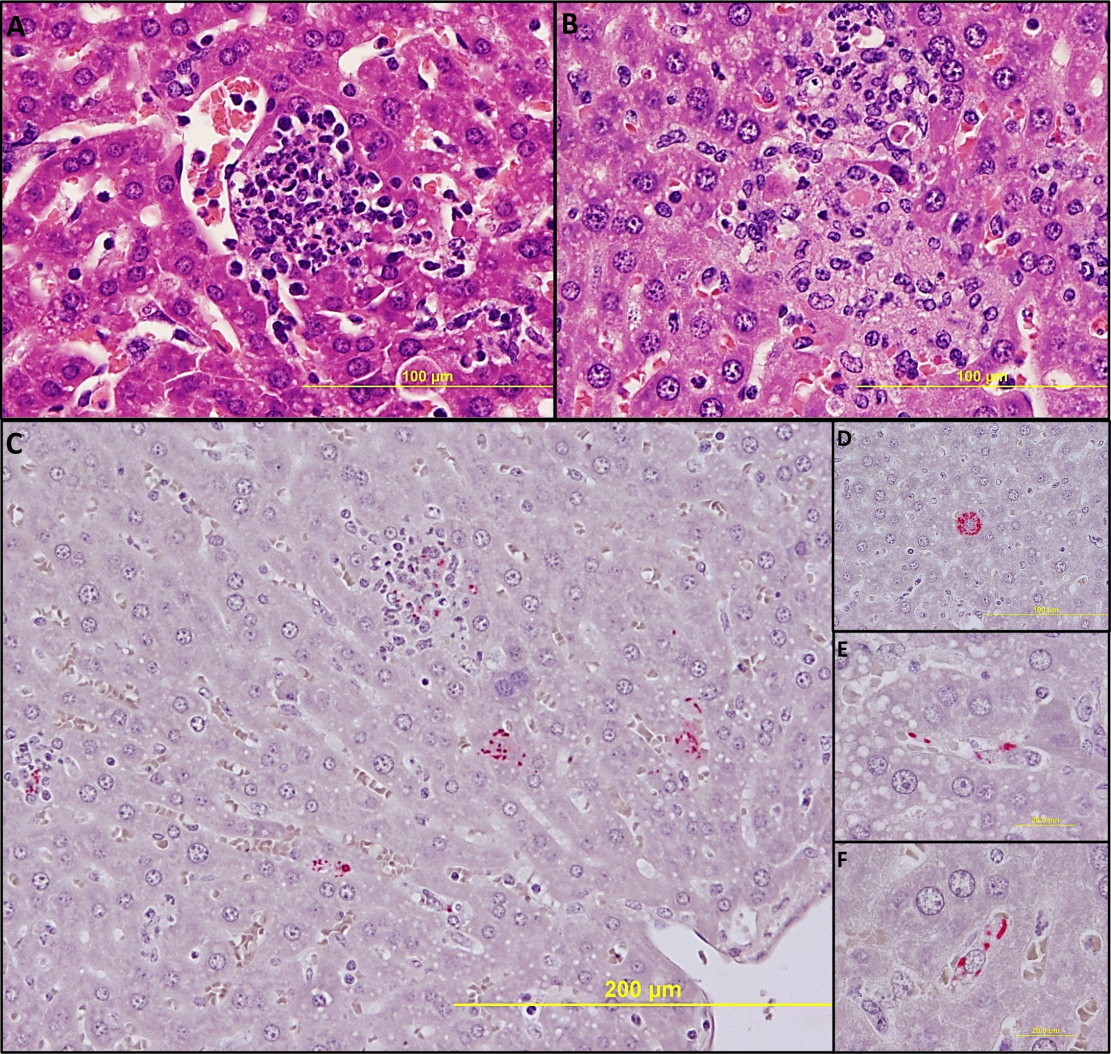
Pathologic lesions in liver. Focal inflammation with polymorphonuclear cell predominance on day 3 p.i. (**A**, 400X), and with mononuclear cell predominance and hepatocyte apoptosis on day 5 p.i. (**B**, 400X). IHC demonstration of SFG rickettsiae in liver: Rickettsiae (red) in multiple types of infected cells (**C**, 200X). In a hepatocyte (**D**, 400X), in endothelial cells (**E**, 1,000X) and in mononuclear cells (**F**, 1,000X) on day 3 p.i.

No significant pathologic findings were detected in the tissues of mice on day 14 after recovery from low- and mid-dose of RpARF or in the uninfected control mice. Meningitis was observed in the brains of animals infected with the high dose of RpARF on days three and five p.i. (Fig 5A and 5B). Pathology in the heart was characterized by mural and valvular endocarditis and perivascular interstitial cellular infiltration beginning on day three (Fig 5E). The kidney showed inflammatory mononuclear cellular infiltration between the renal tubules and intertubular capillaries (Fig 5G). Interstitial pneumonia was observed beginning on day three p.i. (Fig 5I). Cellular infiltration in liver was characterized by a high ratio of polymorphonuclear cells to mononuclear cells on day three p.i., and the inverse was observed on day five p.i., fewer polymorphonuclear cells and an increase in mononuclear cells (Fig 6A and 6B). RpARF organisms were associated with vessels of the microcirculation in areas of pathological damage in the brain, heart, kidney and lung by immunohistochemical staining (Fig 5C, 5D, 5F, 5H and 5J). In the liver, RpARF was observed in hepatocytes and mononuclear cells in addition to endothelial cells (Fig 6C, 6D, 6E and 6F).

## Discussion

To decrease the impact that infectious agents have in public health, valid animal models of infectious diseases are necessary to understand the pathophysiology of the disease and to evaluate vaccine candidates and treatments. Infection of C3H/HeN mice with low- and mid-dose RpARF resulted in dose-dependent severity, whereas infection with the high dose produced a lethal illness. The animals became moribund by day five or six p.i. The lethal disease was characterized by ruffled fur, erythema, labored breathing, decreased activity, and hunched back, which began on day three p.i. and coincided with the peak bacterial loads in some tissues (Fig 4). Other significant observations included splenomegaly (on days three and five p.i.), thrombocytopenia (on days one, three and five p.i.), and neutrophilia (on days three and five p.i.) (Fig 3), which are common findings in murine models of rickettsial infection [25]. Two previous studies established that *R*. *parkeri* causes dose-dependent severity in mice. In one study, the authors infected C3H/HeN mice with 5.5 x 10^6^ of the *R*. *parkeri* Portsmouth strain, and the animals sacrificed on day seven did not develop any clinical signs of illness. The mice displayed mild to moderate splenomegaly, and *R*. *parkeri* DNA was detected in heart and lung of only 50% of the animals [26]. In the other study, 1.2 x 10^7^ *R*. *parkeri* Maculatum 20 strain caused lethargy, mild hunched posture, ruffled fur and decreased activity with onset around days 6–7 p.i. These clinical manifestations persisted for 2–3 days. Bacterial DNA was detected in lung, spleen, liver and brain on day six p.i. [19]. The data from these two reports of C3H/HeN mice infected by intravenous inoculation of *R*. *parkeri* are similar to our low- and mid-dose infections, respectively. But neither of those previous works focused on the development of a lethal model of infection. The focus of one study was the development of a natural mode of transmission using ticks [19]. The objective of the second study was to develop a non-lethal mouse model to study long term progression and the subsequent recovery from SFG rickettsioses [26]. One of the benefits of our model is that it allows for the evaluation of vaccines, therapeutics and immunity against lethal SFG rickettsioses such as Rocky Mountain spotted fever. It is for that reason that the previous studies were only compared with the pilot assay (preliminary dose range experiment). The high dose provides a model that is unique compared with other animal models of *R*. *parkeri* infection.

A study of infection of guinea pigs (*Cavia porcellus*), where the authors used a similar high dose, by intraperitoneal inoculation of 1.9 x 10^8^ *R*. *parkeri* Black Gap strain resulted in a nonlethal illness, manifested in most animals by mild fever and mild to moderate swelling and erythema of the scrotum [3]. This strain has a close genetic identity with *R*. *parkeri* Atlantic Rainforest strain [3,4]. Furthermore, subclinical infection was observed in an additional study that utilized the natural mode of infection of guinea pigs by tick transmission, using *A*. *ovale* nymphs infected with the Brazilian Atlantic Rainforest strain [20].

Various efforts have been undertaken to establish appropriate animal models for *Rickettsia* spp. In 1908, Howard T. Ricketts and L. Gomez described that guinea pigs could be utilized to isolate the agent and to study immunity [27]. Sammons et al. in 1977 demonstrated that infection with *Rickettsia* spp. was lethal in some mouse strains such as Mai:(S) and BALB/cJ infected with 1.3 x 10^6^ plaque forming units (pfu) of *R*. *akari*, Mai:(S) infected with 1 x 10^6^ pfu of *R*. *sibirica*, BALB/cJ infected with 1 x 10^6^ pfu of *R*. *australis* and guinea pig infected with 1 x 10^7^ pfu of *R*. *rickettsii* [28]. Later, in 1984 the model of C3H/HeJ mice infected with 1 x 10^10^ pfu of *R*. *conorii* was described as “an excellent animal model for studying the pathogenesis of *R*. *conorii* infection and for testing the immunogenic potential of experimental rickettsial vaccines” [29]. All these animal models employed intraperitoneal or intradermal inoculation as an infection route and some studies such as a *R*. *conorii* model, utilized a high concentration of bacterial inoculum [27–29]. Currently there are three valid lethal mouse models for rickettsiae that have been characterized, in which the infectious route is intravenous, C57BL/6 or Balb/c mice infected with 2 x 10^6^ pfu of *R*. *australis*, C3H/HeN mice infected with 2.25 x 10^5^ pfu of *R*. *conorii*, and C3H/HeN mice infected with 3 x 10^5^ pfu *R*. *typhi*. All three of these models must be performed in an ABSL-3 laboratory [16–18].

Since this RpARF was isolated in a biosafety level 3 laboratory, all experiments in this work were performed in an animal biosafety level 3 (ABSL-3) laboratory owing to local Institution Biosafety Committee (IBC) restrictions. However, when considering the severity of disease caused by *R*. *parkeri*, it should be noted that it produces a disease less severe than that caused by *Ehrlichia* species, which have been reclassified from BSL3 to BSL2. There is also a precedent for conducting research on *Rickettsia* species at BSL2 [30]. Several low to avirulent species (*R*. *montanensis*, *R*. *rhipicephali*, *R*. *bellii* and *R*. *canadenesis*) can be manipulated at BSL2. It is even recommended that the containment levels of newly discovered rickettsial species should be evaluated on a case by case basis [21,30]. Since preparation of this manuscript we have successfully petitioned our institution to work with this agent in the future at BSL2. Currently, this agent is available in our BSL2 laboratory with the advantage that it can be used in institutions that only have BSL2 laboratories. Since lethal rickettsial animal models only can be performed at BSL3, this model offers an excellent opportunity to work with fewer equipment restrictions, lower costs and greater safety.

In conclusion, we have described an animal model using C3H/HeN mice infected by intravenous inoculation with a 1 x 10^8^ dose of RpARF, which provides an opportunity to study acute lethal disease produced by SFG rickettsiae characterized by infection of endothelial cells in the brain, heart, lung and kidney, and endothelial cells, hepatocytes and mononuclear cells in liver. It is our belief that this experimental infection provides an alternative animal model in mice of rickettsial disease with the advantage that this model can be studied in an animal biosafety level 2 (ABSL-2) laboratory. The benefit of using this at BSL2 is that it will allow for those scientists whose institutions do not have BSL3/ABL3 facilities to be able to conduct research on an actual lethal model of a rickettsial disease and for research equipment that is not available in BSL3 to be utilized.
